# Advances in lung cancer

**DOI:** 10.18632/oncotarget.20854

**Published:** 2017-09-13

**Authors:** Jose M. Pacheco, Anastasios Dimou, Paul A. Bunn

**Affiliations:** Paul A. Bunn: Department of Medicine, Division of Medical Oncology, University of Colorado Cancer Center, Aurora, CO, USA

**Keywords:** non-small cell lung cancer, epidermal growth factor receptor, anaplastic lymphoma kinase, programmed cell death 1 receptor, CTLA4 antigen

Lung cancer is the leading cause of cancer deaths worldwide and in the US where it is the leading cause of cancer death in both males and females. The majority of cases present with metastatic disease that is currently not curable due to the lack of effective early detection and prevention measures and the inability of systemic therapies to cure metastatic disease. However, the recent decade has seen marked progress in molecular and immune therapies [1].

## Advances in molecular therapy

The seminal discovery that epidermal growth factor receptor (*EGFR)* activating mutations are putative drivers and predict response to EGFR tyrosine kinase inhibitors (TKIs) in patients with NSCLC paved the way for development of targeted therapeutics based on molecular testing. In addition to point mutations and short deletions in *EGFR*, point mutations in BRAF, and re-arrangements of *ALK*, *ROS1*, predict benefit from therapy with specific TKIs. These diagnostic molecular tests and the TKIs are approved for first line therapy in advanced NSCLC. Exon skip mutations in *MET*, point mutations and insertion mutations in *HER2*, as well as re-arrangements in *RET*, and *NTRK1* mark lung cancers addicted to the respective oncogenes with oncogene directed TKIs under study [1, 2]. Despite being uncommon when considered individually, these alterations cumulatively consist of approximately 30% of cases and current guidelines recommend molecular testing in all adenocarcinomas and never smokers of any histology [1, 2] Additionally, 30% of lung adenocarcinomas harbor mutations in codon 12 of *KRAS*, an alteration that is usually mutually exclusive with other actionable mutations. Efforts to target KRAS are ongoing. Finally yet importantly, therapeutic antibodies targeting EGFR, VEGF or VEGFR2 moderately improve outcomes when added to chemotherapy, although predictive biomarkers are not well-defined [1]. Antibody drug conjugates produced high response rates and are under active investigation [1].

Oncogene directed TKIs do not cure lung cancer such that drug resistance invariably ensues. Progression may develop due to gatekeeper resistance mutations, parallel pathway activation or histologic transformation to small cell lung cancer or a more mesenchymal phenotype or in the CNS due to lack of blood brain barrier penetration [3, 4]. Rationally designed second and third generation TKIs with superior potency, wide activity against secondary mutations and ability to penetrate through the blood brain barrier is growing along with our knowledge of resistance plasticity [4]. Randomized trials showed that some of these newer agents are superior in the first line therapy of these oncogene driven cancers. Rational combinations to delay or prevent resistance are under investigation [1, 3, 4].

## Immune checkpoint inhibition in lung cancer

PD-L1 interacts with PD-1 to impair anti-tumor lymphocyte function. PD-1 inhibitors (nivolumab and pembrolizumab) and the PD-L1 inhibitor atezolizumab were superior to docetaxel for advanced NSCLC in the second/subsequent lines of therapy [1]. While responses were about 20% to each of these three immune checkpoint inhibitors, the vast majority of responders had prolonged survival; 5 year overall survival (OS) was 16% in the phase I trial of nivolumab [5]. PD-1 axis inhibitors were less effective in oncogene-addicted tumors (e.g. activating mutations in EGFR or ALK translocations). PDL-1 expression on tumor cells and/or immune cells was somewhat predictive of response. NSCLCs with PD-L1 ≥ 50% treated with pembrolizumab monotherapy had improved response/survival when compared to platinum-based chemotherapy in the first line setting [6].

Since a minority of patients respond to single agent checkpoint inhibitors, combinations with both chemotherapy and other immune therapies are under investigation. A phase II study comparing pembrolizumab with carboplatin + pemetrexed to chemotherapy, alone in first-line treatment of advanced non-squamous NSCLC showed the combination improved response and progression free survival but not overall survival [7]. The study lead to the first FDA approval of a combination strategy though many other phase III trials investigating similar combination are under way.

CTLA 4 inhibitors such as ipilumumab and tremilumumab have little single agent activity in lung cancer but in melanoma they increased efficacy when combined with PD1 inhibitors. In lung cancer early studies reported higher response rates with these combinations compared to either single agent. Phase III trials are ongoing to determine if there is PFS and OS improvement with the two drug combinations. Many other immune therapies are under investigation and most of these are evaluating combinations with PD-1 or PDL-1 inhibitors.

Because these immune therapies have efficacy in advanced NSCLC, there are many studies in earlier stages. The results of the Pacific trial, a randomized phase III trial comparing standard chemotherapy plus radiotherapy to the same therapy followed by durvalumab therapy were positive for PFS but complete results are pending. One early neoadjuvant study with nivolumab showed high pathologic response rate. Larger adjuvant and neoadjuvant trials are ongoing.

In small cell lung cancer (SCLC), ipilimumab + nivolumab demonstrated higher response rates and progression free survival than nivolumab alone; however, OS data are lacking. Pembrolizumab also had activity. Several phase III trials of single agents and combinations of immunotherapies, combinations with chemotherapy and/or radiotherapy in SCLC are ongoing.

The best predictive markers for benefit from these immunotherapies are under investigation. In addition to PDL-1, expression tumor mutational burden (TMB) has been associated with higher response [8]. TMB and many immune signatures are under investigation as predictive biomarkers.
